# Evidence Based Alternative Medicines in Pain Management 2016

**DOI:** 10.1155/2016/7078351

**Published:** 2016-09-25

**Authors:** Haroon Khan, Vincenzo De Feo, Najeeb Ur Rehman, Agnieszka Najda

**Affiliations:** ^1^Department of Pharmacy, Abdul Wali Khan University, Mardan 23200, Pakistan; ^2^Department of Pharmacy, University of Salerno, Fisciano, 84084 Salerno, Italy; ^3^UoN Chair of Oman's Medicinal Plants and Marine Natural Products, University of Nizwa, Oman; ^4^Quality Laboratory of Vegetable and Medicinal Materials, Department of Vegetable Crops and Medicinal Plants, University of Life Sciences in Lublin, 58 K. St. Leszczyńskiego Street, 20-068 Lublin, Poland

Pain is an unpleasant sensation but indeed it is one of the vital alarm systems in human body. It helps in the recognition of various stimuli of intensities that could be potentially harmful to the tissue. In comparison to short-lasting acute pain, chronic pain persists even after the resolution of the initial cause and often loses its protective value, rather producing unwanted effects. Pain of any origin, if it persists, may become resistant to standard treatments, greatly affecting the patients' quality of life. Therefore, advancing our understanding of the pathogenesis of chronic pain is crucial to identify novel therapeutic approaches. There are several therapeutic approaches which are used for the effective management of pain including use of drugs and alternate measures.

The use of medicinal plants in the management of pain is as old as man himself. In this connection, G. R. Donald et al. investigated the antinociceptive activity of* Zanthoxylum piperitum* DC essential oil in various animal models. The oil showed marked antinociceptive effect in formalin-induced acute pain model and glutamate-induced nociception. However, capsaicin-induced nociception and carrageenan induced paw edema were not antagonized by the* Z*.* piperitum* oil. Similarly, a Malaysian research group studied the antinociceptive effect of methanolic extract of* Clinacanthus nutans* leaves* in vivo*. After producing significant pain reliving effects in acetic acid induced abdominal constriction, formalin-induced paw licking, and hot-plate tests, extract was subjected to the determination of possible mechanism. This antinociceptive activity was fully antagonized by naloxone (a nonselective opioid antagonist) but was partially reversed by L-arginine (L-arg; a nitric oxide [NO] precursor), N*ω*-nitro-L-arginine methyl ester hydrochloride (L-NAME; NO synthase inhibitor), or their combinations thereof.

E. D. Stolz et al. evaluated the uliginosin B (ULI) a natural acylphloroglucinol that has been proposed as a new molecular scaffold for developing analgesic and antidepressant drugs. Its effect is generally attributed to its ability to increase monoamines in the synaptic cleft by inhibiting their neuronal uptake without binding to their respective transporters. Additionally, the selective adenosine A receptor antagonist DPCPX and the selective A 2A antagonist ZM-241385 prevented its effect in the hot-plate test in mice. Pretreatment with inhibitors of adenosine reuptake (dipyridamole) or adenosine deaminase (EHNA) did not affect the ULI effect. On the other hand, its effect was completely prevented by an inhibitor of ecto-5′-nucleotidase (AMPCP). This finding was confirmed* ex vivo*, whereby ULI treatment increased AMP and ATP hydrolysis in spinal cord and cerebral cortex synaptosomes, respectively. Thus, the activation of A_1_ and A 2A receptors and the modulation of ecto-5′-nucleotidase activity contributed to the antinociceptive to its effect. Y.-J. Qu et al. showed mitogen-activated protein kinases (MAPKs); pathways were involved in neuropathic pain in rats with chronic compression of the dorsal root ganglion. The specific inhibitors of MAPKs contributed to the attenuation of mechanical allodynia CCD rats and the large size MAPKs positive neurons in dorsal root-ganglia were crucial.

The Traditional Chinese medicine (TCM) has developed and used a sophisticated system of individualized medicine in the form of pattern diagnosis and classification for hundreds years. Acupuncture is a centuries old practice in the traditional Chine's system of treatment for pain management. The study used acupuncture treatment for lateral elbow pain (LEP) as an example to study the diagnostic practice of individualized acupuncture treatment. A provisional version of LEP pattern questionnaire was developed based on a recent systematic review on TCM pattern diagnosis for LEP. A Delphi panel of 33 clinical experts from seven different countries was formed in Chinese and English language. Consensus was found on four TCM patterns that could underlie LEP, namely, the* wind-cold-dampness pattern*, the* qi stagnation and blood stasis pattern*, the* dual deficiency of qi and blood pattern*, and the* retained dampness-heat pattern*. A list of signs and symptoms indicating one of the four TCM patterns and a list of preferred treatment modalities for each pattern were also generated.

The systemic review and meta-analysis of Z. Feng et al. on the efficacy and safety of the combination of total glucosides of peony and leflunomide for the treatment of rheumatoid arthritis concluded that the combination of total glucosides of peony and leflunomide in treatment of RA presented the characteristics of notably decreasing the levels of laboratory indexes and higher safety in terms of liver function for the treatment of rheumatoid arthritis. However, this conclusion should be further investigated by increasing the sample size.

Cupping therapy (CT) is a traditional Chinese medical (TCM) treatment which has been practiced for thousands of years. Researchers from Taiwan investigated the effectiveness of cupping therapy (CT) in changes on skin surface temperature (SST) for relieving chronic neck and shoulder pain (NSP) among community residents. A quasi experimental design consists of sixty subjects with self-perceived NSP. The results showed that no participants experienced localized skin burns or adverse reactions in the treatment regions. Two participants in the cupping group reported mild low back pain related to the seated position. In this study, one treatment of CT is shown to increase SST and reduce SBP. In conjunction with the physiological effects, the subjective experience of NSP is reduced. CT mimics an analgesic effect which has no known negative side effects and may be considered safe. However, further studies are required to improve the understanding and potential long term effects of CT.

In conclusion, pain management is still a major clinical problem worldwide. The different articles of this special issue indicated that various researchers around the world are working on both chemical and nonchemical interference for effective pain management in order to get patient compliance. We can also suggest that the combination of drugs with other techniques (without use of drugs) could provide better results in painful conditions.

*Haroon Khan* *Haroon Khan* *Vincenzo De Feo* *Vincenzo De Feo* *Najeeb Ur Rehman* *Najeeb Ur Rehman* *Agnieszka Najda* *Agnieszka Najda*

